# Detection of PIK3/AKT pathway in Moroccan population with triple negative breast cancer

**DOI:** 10.1186/s12885-018-4811-x

**Published:** 2018-09-18

**Authors:** Farah Jouali, Nabila Marchoudi, Salwa Talbi, Basma Bilal, Mohamed El Khasmi, Houria Rhaissi, Jamal Fekkak

**Affiliations:** 1 Anoual Laboratory of Radio-Immuno Analysis, Angle Blvd Alexandrieet Blvd Anoual, 20360 Casablanca, Morocco; 2 Laboratory of Pathophysiology and Molecular Genetics, Ben M’Sik Faculty of Science, 7955 Casablanca, Morocco; 3 Department of Oncology, Center Hospital University Mohammed VI, 40080 Marrakech, Morocco; 4 Department of Pathology, Center Hospital University Ibn Rochd, 20100 Casablanca, Morocco

**Keywords:** *PIK3CA*, *AKT*, *PTEN*, Triple negative breast cancer, PI3K pathway

## Abstract

**Background:**

Triple Negative Breast Cancer (TNBC) is an aggressive form of breast cancer, that represents 10–20% of all breast carcinomas and characterized by the lack of a specific cell surface marker compared to other breast cancer subtypes. Due to the absence of molecular markers for TNBC his treatment options remains limited, without proven targeted therapies, which emphasize the need for discovering molecular markers that could be targeted for patient treatment, An important number of TNBC cases harbor aberrations in the phosphoinositide 3-kinase (PI3K) pathway, leading to constitutive activation of the downstream signaling pathway. Among mechanisms of PI3K enhancement, *PIK3CA* mutations are most frequently (~ 30%) observed, along with protein loss of *PTEN* and *AKT* activation by phosphorylation (pAkt). Therefore, we propose to analyze clinocopathologic and molecular characteristics of PI3K/AKT/PTEN pathway in Moroccan triple negative breast cancer patients.

**Methods:**

We conducted a retrospective study of 39 patients diagnosed with triple negative breast cancer between early 2013 and 2016. In this study, we used the Ion Personal Genome Machine (PGM) and Ion Torrent Ampliseq Cancer panel to sequence hotspot regions from PIK3CA, *AKT* and *PTEN* genes to identify genetic mutations in 39 samples of TNBC subtype from Moroccan patients and to correlate the results with clinical-pathologic data.

**Results:**

All patients were female with a median age of 46 years from (34–65). Most patients have had invasive ductal carcinoma (84.6%) and 69.2% of them were grade III SBR. Among the 39, 9 were right sided tumor patients and the remaining 30 were left-sided. Mutational analysis of PIK3CA gene was achieved in all TNBC patients. PIK3CA hotspot mutations were detected in 5/39 of TNBC (13%), in detail, among these 5 TNBC patients, one harbored mutation in exons 9 and four in exon 20.

**Conclusion:**

The PI3KCA gene is highly activated and plays a crucial role in the pathogenesis of TNBC more, therefore, may be a potential therapeutic target to improve outcomes in patients.

## Background

Triple-negative breast cancer (TNBC) is a highly diverse group of cancers defined by the lack of oestrogen, progesterone, and ERBB2 receptors expression, it may account for 10–20% of all newly diagnosed breast cancer cases [1], and it mostly arises in younger and premenopausal women [2]. TNBC is generally associated with larger size, higher histological grade, more advanced disease stage and a tendency towards local and visceral metastases showing the worse prognosis compared to other breast cancer subtypes [3, 4] .

Although many reports claim that TNBCs respond to chemotherapy better than other types of breast cancer, his prognosis remains poor [3] and this is due to shortened disease-free interval in the adjuvant and neoadjuvant setting and a more aggressive course in the metastatic setting [4]. Therefore, establishing a new effective molecular therapeutic approach in killing TNBC cells is crucially required to improve TNBC patients outcome [5]. This emphasize the importance of molecular studies in this breast cancer subtype.

Since the development of molecular technologies tell now many genomic studies has been conducted in purpose of better understanding the molecular nature of triple negative breast cancer, these studies have shown that TNBC could be classified into numerous independent subtypes remarkably heterogeneous at the genomic level [6], They further shed light into the large number of genes and major cellular pathways potentially involved in TNBC tumorigenesis. In the last decade, there has been intensive research to define the relative contributions of these genes and cellular pathways in TNBC and to identify therapeutic targets for TNBC based on genomics [7].

The PI3K/AKT is one of the most frequently altered pathway in breast cancer.in TNBC is the second most altered after TP53 gene. This pathway is implicated in many cell cycle process like cell survivor or proliferation [8], these process are ensured by serine and/or threonine phosphorylation of this pathway downstream substrates [9]. The key proteins involved are phosphatidylinositol 3-kinase (PI3K) and Akt/Protein Kinase B. PI3K protein is an heterodimer consists of two subunits: p110 catalytic subunit encoded by PIK3CA (p110α), PIK3CB (p110β) or PIK3CD (p110δ) and one p85 regulatory subunit encoded by PIK3R1 (p85α), PIK3R2 (p85β) or PIK3R3 (p85γ) [10]. This protein is responsible for the phosphorylation of Akt, a serine/threonine kinase through the phosphorylation of phosphatidylinositol 4,5 bisphosphate (PIP2), to phosphatidylinositol 3,4,4-triphosphate (PIP3). The phosphorylation of AKT initiate a downstream signaling cascade that is regulated by The tumor suppressor Phosphatase and tensin homolog deleted on chromosome ten (PTEN) this protein reverses the effects of PI3K by dephosphorylating the same site on membrane phosphatidylinositols that is phosphorylated by PI3K [11, 12]. In cancer genomic alterations in one of this pathway key proteins like gene mutations or duplications cause cell cycle dysregulation leading to cancer genesis, and because genomic aberrations can predict responsiveness to targeted therapies, and because multiple PI3K pathway members are frequently aberrant in breast tumors, targeting this pathway may provide a highly effective therapeutic approach [13, 14].

PIK3CA mutations are the most common genetic alteration of this pathway. Approximately 80% of PIK3CA somatic mutations are located in two common hotspot regions [15], the helical that contains E542K or E545K mutations in exon 9 and the kinase that carries H1047R or H1047L mutation in exon 20. The mechanism by which they promote constitutive PI3K signaling remains unclear [16]. Both types of mutation were indicated to be gain-of-function and transforming activity [17, 18].

On the other hand, genomic aberrations observed in the PI3K pathway in breast cancer include also activating alterations, involving the AKT and loss/mutation of PTEN [19, 20].The prevalence of AKT1 mutation (E17K) in breast cancer is 8%, which has been shown to lead to notable kinase activity than that of wild-type AKT1 [21]. Therefore, AKT integrates various upstream inputs and triggers downstream network activities [17].While PTEN mutations are relatively uncommon in breast cancer (< 5%), PTEN protein loss is frequent (~ 30%) [12, 22].This loss is reported to be caused by various mechanisms, such as promoter methylation, loss of heterozygosity, and regulation at the RNA or protein level. [12, 22, 23]

The current study aim to analyze the clinocapathologic and molecular characteristics of PI3K/AKT/PTEN pathway in Moroccan triple negative breast cancer patients.

## Patients and methods

### Patients

The current *study* involved TNBC *patients* diagnosed at the University Hospital Center of Casablanca and Marrakech, Morocco, between early 2013 and 2016. The primary inclusion criterion was an adequate fresh tumor obtained from a resected tumor sample. The enrolled patients met the following criterion: (I). Patients with pathologically proved TNBC. (II). Cases with the complete clinical, pathological and follow-up data. (III) Patients between the age of 20 and 65. Informed consent was obtained from patients to use their surgical specimens and clinic-pathological data for research purposes.

This retrospective study was performed at Anoual Laboratory of Radio-Immuno Analysis, Casablanca, Morocco, where the samples were processed for NGS.

### Methods

#### DNA extraction

Tumors consisting of over 50% malignant cells were dissected under microscopy from 4-mm unstained sections by comparison with a hematoxylin and eosin (HE) stained slide, and genomic DNA was extracted using a Qiagen DNA FFPE Tissue kit (Qiagen, Hilden, Germany) according to the manufacturer’s instructions. After extraction, DNA concentration was measured using the Qubit dsDNA HS (High Sensitivity) Assay kit and the Qubit® Fluorometer (Invitrogen; Thermo Fisher Scientific, Inc., Waltham, MA, USA).

#### Library preparation

Ten nanograms of DNA was used for preparing amplicon library using Ion AmpliSeq™ Library kit 2.0 (Ion Torrent; Thermo Fisher Scientific, Inc.) and Ion AmpliSeq™ Cancer hotspot panel (Ion Torrent; Thermo Fisher Scientific, Inc.), according to manufacturer’s instructions. This panel contains 207 primer pairs in a single tube and surveys hotspot regions of 50 oncogenes and tumor suppressor genes. The genes included in the panel were: ABL1, AKT1, ALK, APC, ATM, BRAF, CDH1, CDKN2A, CSF1R, CTNNB1, EGFR, ERBB2, ERBB4, EZH2, FBXW7, FGFR1, FGFR2, FGFR3, FLT3, GNA11, GNAS, GNAQ, HNF1A, HRAS, IDH1, JAK2, JAK3, IDH2, KDR, KIT, KRAS, MET, MLH1, MPL, NOTCH1, NPM1, NRAS, PDGFRA, PIK3CA, PTEN, PTPN11, RB1, RET, SMAD4, SMARCB1, SMO, SRC, ST11, TP53 and VHL. Each library was barcoded using Ion Xpress™ Barcode Adapters kit (Ion Torrent; Thermo Fisher Scientific, Inc.).

#### Template preparation by emulsion polymerase chain reaction (PCR)

The amplified and purified libraries were diluted to a final concentration of 100 pM. Template preparation by emulsion PCR was carried out using the Ion PGM™ Template Hi-Q OT2 200 kit, followed by the Ion OneTouch™ 2 System (Ion Torrent; Thermo Fisher Scientific, Inc.), according to manufacturers’ protocol.

The polyclonal percentage and quality of the enriched, template-positive ISPs was determined using the Ion Sphere Quality Control Kit (Ion Torrent; Thermo Fisher Scientific, Inc.). The optimal amount of template-positive ion sphere particles (ISPs) is 10–30%. ISPs were then enriched for template positive ISPs using Dynabeads MyOne Streptavidin C1 beads (Invitrogen; Thermo Fisher Scientific, Inc.) in the Ion OneTouch™ ES instrument (Ion Torrent; Thermo Fisher Scientific, Inc.).

#### Ion torrent PGM sequencing

Finally, sequencing was carried out on the Ion Personal Genome Machine System PGM (Ion Torrent; Thermo Fisher Scientific, Inc.) using Ion 316™ Chips and the Ion PGM™ Sequencing Hi-Q Kit v2, according to the manufacturer’s guidelines. For a sequence variant to be considered authentic, a sequencing coverage of 100X reads was used as a minimum requirement in the present study.

#### Bioinformatics and statistical analysis for AmpliSeq

*Data* from the sequencing runs *were* initially *processed* Torrent Suite software v5.4 (Ion Torrent; Thermo Fisher Scientific, Inc.) to generate *sequence* reads, filter, and remove poor signal-profile reads using the reference genomic sequence (hg19) of target genes.

Coverage analysis and variant calling was performed with Torrent Variant Caller plugin software v5.4. Following data analysis, annotation of single-nucleotide variants, insertions, deletions and splice site alterations was performed using the Ion Reporter Server System (Thermo Fisher Scientific, Inc.) and the Ingenuity® Variant Analysis Software (Qiagen, Inc.).

### Statistical analysis

Statistical analysis of clinic-pathologic data was performed using IBM SPSS Statistics ver. 21 (IBM Co., Armonk, NY). Chi-square (χ2) test and Fisher’s exact test were performed to assess significance of the association between variables (PIK3CA, AKT and PTEN mutations in TNBC patients) and clinical characteristics. Statistical difference was defined as *P* < 0.05.

## Results

In total, 47 TNBC patients were enrolled in this study. Of the 47 patients, targeted DNA sequencing was performed on 39 patients only. Samples from 8 patients did not undergo next generation sequencing due to DNA extraction failure. TNBC samples were characterized by ER, PR, and HER2 negativity, with ki67 proliferation index ranging from 30 to 90%. Clinico-pathological characteristics of the patients are summarized in Table 1. All patients were female with a median age of 46 years from (34–65). Among the 39, 9 were right sided tumor patients and the remaining 30 were left-sided. Most patients were invasive ductal carcinoma and had high histological grade (grade III). The clinical-pathologic data of TNBC according to mutational status of PIK3CA included in this study are also reported in Table 1.

**Table 1:** Clinic-pathologic and biologic data of the TNBC patients according to mutational status of PIK3CA

	Variables	Overall		PIK3CA wild type		PIK3CA mutated		*P*-value
		effective	%	effective	%	effective	%	
Age range	< 40	11	28.2	9	23.07	2	5.1	0.542
	> 40	28	71.7	25	64.1	3	7.6	
Localization	Left breast	30	76.9	25	64.1	5	12.8	0.09
	Right breast	9	23.0	9	23.1	0	0	
Histopathologic type	Ductal carcinoma invasive	33	84.6	29	74.3	4	10.2	0.729
	Lobular carcinoma invasive	6	15.3	5	12.8	1	2.5	
Grade SBR	II	12	30.7	10	25.6	2	5.1	0.639
	III	27	69.2	24	61.5	3	7.6	
Ki67	< 20%	0	0	0	0	0	0	0.254
	20–50%	8	20.5	6	15.3	2	5.1	
	> 50%	31	79.4	28	71.7	6	15.3	

### Mutational analysis of PIK3CA, AKT and PTEN gene

Mutational analysis of PIK3CA gene was achieved in all TNBC patients. PIK3CA hotspot mutations were detected in 5/39 of TNBC (13%), in detail, among these 5 TNBC patients, oneharbored mutation in exons 9 and four in exon 20.

Apropos the analysis of PIK3CA exon 9, involving the helical domain of PIK3CA gene, our results showed the presence of one somatic mutation c.1633G > A p.E545K, which was revealed in 1/39 TNBC (2.5%). On the other hand, the mutational analysis of PIK3CA exon 20, involving the kinase domain of PIK3CA gene, showed the presence of one hotspot mutation c.3140A > G p.H1047R, which was identified in 4/39 TNBC (10.2%). No case had mutations in both exons and coexistence of two or more mutations. In contrast, no AKT1 and PTEN mutations were detected in 39 patients that were successfully sequenced.

The correlation of PIK3CA alteration status with clinical data was investigated in TNBC patients. (Table 1). In short, there were no associations between mutational status of PIK3CA with age (*p* = 0.542), histopathological type (*p* = 0.09), localization (*p* = 0.729) or histological grade (*p* = 0.639). No significant associations were found between PIK3CA mutational status and clinico-pathological features according to TNBC subgroup.

## Discussion

The phosphatidylinositol 3-kinase (PI3K/Akt) signaling cascade is crucial to divergent physiological processes, which include cell cycle progression, differentiation, and metabolism [24]. The PI3K/Akt pathway aberrations are common in breast cancer, pointing to a critical role for this signaling pathway in breast carcinogenesis [22]. This pathway is frequently activated in triple negative breast cancer (TNBC), via molecular abnormalities such as *PIK3CA* mutations or loss of PTEN function.As has been observed previously, PIK3CA oncogene mutations were more common in TNBC unlike Akt1 and PTEN mutations [25–27].

In the present study, the mutation rate of PIK3CA in triple negative breast cancer was 13% (5/39). Two different PIK3CA mutations were identified: c.1633G > A p.E545K in helical domain encoded by exon 9 and c.3140A > G p.H1047R in the kinase domain encoded by exon 20. Mutations p.E545K and p.H1047Rhave been found in previous studies to be the most prevalent in breast cancer and are associated with an increase activity in the PI3K pathway. These findings are consistent with those of Chen YH et al. study, which have found a prevalence of 16% (6/38) [28]. This was confirmed by Milis et al. study comprising a larger cohort of 702 TNBC patientsin whom 93 had mutations in the PIK3CA gene (ie 13%) [26].

The rate obtained in our study remains lower than that reported by Cossu-Rocca et al., who in a cohort of 97 TNBC patients, 23% were found to have PIK3CA mutation [29]. This rate were further supported by Kriegsmann et al. who showed 22,1% PIK3CA mutation rate in a cohort of 104 TNBC patients [30].

These differences in rates can be explained by the difference in the studied populations, particularly African-Americans who have a low percentage of PI3KCA gene mutations compared with the Caucasian population. This hypothesis was observed in the study of Ademuyiwaet al [31].

We were interested in testing the relationship between PIK3CA mutational status and other known clinic-pathologic markers (table1). No association was identified regarding PIK3CA mutations and patient age, histopathological type or grade of the tumors. These results are similar to those of Paul S Weisman et al. [32].

Besides PIK3CA gene, AKT1 and PTEN gene also known to be altered in PIK3 pathway. In the present study, no mutations were detected in AKT1 and PTEN genes. These results are consistent with those of Stemke-Hale et al. who in a cohort of 20 TNBC tumors showed no mutation in PTEN gene [22]. Furthermore, the same study also investigated the AKT1 E17K mutation prevalence in a cohort of 111 TNBC patients and found no mutations [22].Another study performed by K. Hashimoto et al., confirm our results since no mutations was detected in 75 TNBC patients [33].

Mutations in the PTEN gene are rarely seen in breast cancers (< 5%), however the loss of expression of PTEN protein (promoter methylation, loss of heterozygosity) is widespread [10]. Expression analysis of PTEN by immunohistochemistry revealed a loss of expression in 69% of triple negative tumors (372 cases) according to a study by Millis et al. [26] and 77% of cases according to the study by Shaham Beg et al. [34].

Mutations of the AKT1 gene have been reported in 1.4 to 8% (mean ~ 4%) of infiltrating ductal carcinomas [22, 35]. The E17K mutation of the AKT1 gene is considered a potential diagnostic biomarker for breast cancer [36], which led us to search for this mutation in our study. However, no mutations were detected and this was supported by two large sequencing studies who also failed to find mutations in the Akt1 gene [37, 38].

The absence of mutations in Akt1 and PTEN genes does not necessarily means that there is not any other alterations in epigenetics that might cause problems in the protein. Therefore, PTEN and AKT expression and post-transcriptional status in TNBC patients may be explored.

The PI3K-AKT-mTOR pathway is clearly a fundamental pathway involved in carcinogenesis (proliferation, apoptosis, differentiation and neo-angiogenesis). The presence of multiple mediators in the PI3K signaling pathway means that there are several specific targets to achieve cell arrest and to limit the toxicity of therapies [39].

In recent years, several inhibitors have emerged and have been the subject of clinical trials. Use of PI3K pathway inhibitors as single-agent therapies has proved minimally effective in somediseases [40]. The complexity of the PI3K pathway with multiple feedback mechanisms explains some of the drug resistance. Combination drug therapies that simultaneously target these escape mechanisms will likely lead to the greatest clinical success [41]. The first drugs entering the clinical trials were the inhibitors of mTOR (Rapamycin) and its analogues [40].

## Conclusion

TNBC is a heterogeneous subtype. Its complexity at the clinical and genetic levels illustrates that no single treatment approach will produce universal benefit in this disease.The PI3K/AKT pathway is highly activated and plays a crucial role in the pathogenesis of TNBC and, therefore, may be a potential therapeutic target to improve outcomes in patients.

## Abbreviations

ER: Estrogen Receptor
FFPE: formalin-fixed paraffin-embedded
HE: hematoxylin and eosin
HER-2: Human Epidermal Growth Factor Receptor 2
HS: High Sensitivity
mTOR: mechanistic target of rapamycin
OT2: One Touch 2
PGM: Personal Genome Machine
PI3K: Phosphoinositide 3-kinase
*PIK3CA*: Phosphatidylinositol-4,5-Bisphosphate 3-Kinase Catalytic Subunit Alpha
PR: Progesterone Receptor
PTEN: Phosphatase and Tensin homolog deleted on chromosome ten
TNBC: Triple Negative Breast Cancer
